# Quantum machine learning for electronic structure calculations

**DOI:** 10.1038/s41467-018-06598-z

**Published:** 2018-10-10

**Authors:** Rongxin Xia, Sabre Kais

**Affiliations:** 1 Department of Physics and Astronomy, Purdue University, West Lafayette, IN 47907 USA; 2 Department of Chemistry and Birck Nanotechnology Center, Purdue University, West Lafayette, IN 47907 USA; 3 Santa Fe Institute, 1399 Hyde Park Rd, Santa Fe, NM 87501 USA

## Abstract

Considering recent advancements and successes in the development of efficient quantum algorithms for electronic structure calculations—alongside impressive results using machine learning techniques for computation—hybridizing quantum computing with machine learning for the intent of performing electronic structure calculations is a natural progression. Here we report a hybrid quantum algorithm employing a restricted Boltzmann machine to obtain accurate molecular potential energy surfaces. By exploiting a quantum algorithm to help optimize the underlying objective function, we obtained an efficient procedure for the calculation of the electronic ground state energy for a small molecule system. Our approach achieves high accuracy for the ground state energy for H_2_, LiH, H_2_O at a specific location on its potential energy surface with a finite basis set. With the future availability of larger-scale quantum computers, quantum machine learning techniques are set to become powerful tools to obtain accurate values for electronic structures.

## Abstract (web summary)

With the rapid development of quantum computers, quantum machine learning approaches are emerging as powerful tools to perform electronic structure calculations. Here, the authors develop a quantum machine learning algorithm, which demonstrates significant improvements in solving quantum many-body problems.

## Introduction

Machine learning techniques are demonstrably powerful tools displaying remarkable success in compressing high dimensional data^1,2^. These methods have been applied to a variety of fields in both science and engineering, from computing excitonic dynamics^3^, energy transfer in light-harvesting systems^4^, molecular electronic properties^5^, surface reaction network^6^, learning density functional models^7^ to classify phases of matter, and the simulation of classical and complex quantum systems^8–14^. Modern machine learning techniques have been used in the state space of complex condensed-matter systems for their abilities to analyze and interpret exponentially large data sets^9^ and to speed-up searches for novel energy generation/storage materials^15,16^.

Quantum machine learning^17^—hybridization of classical machine learning techniques with quantum computation—is emerging as a powerful approach allowing quantum speed-ups and improving classical machine learning algorithms^18–22^. Recently, Wiebe et al.^23^ have shown that quantum computing is capable of reducing the time required to train a restricted Boltzmann machine (RBM), while also providing a richer framework for deep learning than its classical analog. The standard RBM models the probability of a given configuration of visible and hidden units by the Gibbs distribution with interactions restricted between different layers. Here, we focus on an RBM where the visible and hidden units assume {+1,−1} forms^24,25^.

Accurate electronic structure calculations for large systems continue to be a challenging problem in the field of chemistry and material science. Toward this goal—in addition to the impressive progress in developing classical algorithms based on ab initio and density functional methods—quantum computing based simulation have been explored^26–31^. Recently, Kivlichan et al.^32^ show that using a particular arrangement of gates (a fermionic swap network) is possible to simulate electronic structure Hamiltonian with linear depth and connectivity. These results present significant improvement on the cost of quantum simulation for both variational and phase estimation based quantum chemistry simulation methods.

Recently, Troyer and coworkers proposed using a restricted Boltzmann machine to solve quantum many-body problems, for both stationary states and time evolution of the quantum Ising and Heisenberg models^24^. However, this simple approach has to be modified for cases where the wave function’s phase is required for accurate calculations^25^.

Herein, we propose a three-layered RBM structure that includes the visible and hidden layers, plus a new layer correction for the signs of coefficients for basis functions of the wave function. We will show that this model has the potential to solve complex quantum many-body problems and to obtain very accurate results for simple molecules as compared with the results calculated by a finite minimal basis set, STO-3G. We also employed a quantum algorithm to help the optimization of training procedure.

## Results

### Three-layers restricted Boltzmann machine

We will begin by briefly outlining the original RBM structure as described by^24^. For a given Hamiltonian, *H*, and a trial state, $|\phi \rangle = \mathop {\sum}\nolimits_x \phi (x)|x\rangle$, the expectation value can be written as:^24^

1 $$\langle H\rangle 	= \frac{{\langle \phi |H|\phi \rangle }}{{\langle \phi |\phi \rangle }} = \frac{{\mathop {\sum}\nolimits_{x,x\prime } {\langle \phi |x\rangle \langle x|H|x\prime \rangle \langle x\prime |\phi \rangle } }}{{\mathop {\sum}\nolimits_x {\langle \phi |x\rangle \langle x|\phi \rangle } }} \\ 	= \frac{{\mathop {\sum}\nolimits_{x,x\prime } {\overline {\phi (x)} \langle x|H|x\prime \rangle \phi (x\prime )} }}{{\mathop {\sum}\nolimits_x {|\phi (x)|^2} }}$$

where *ϕ*(*x*) = 〈*x*|*ϕ*〉 will be used throughout this letter to express the overlap of the complete wave function with the basis function |*x*〉, $\overline {\phi (x)}$ is the complex conjugate of *ϕ*(*x*).

We can map the above to a RBM model with visible layer units $\sigma _1^z,\,\sigma _2^z...\,\sigma _n^z$ and hidden layer units *h*_1_, *h*_2_... *h*_*m*_ with $\sigma _i^z$, *h*_*j*_ ∈ {−1, 1}. We use a visible unit $\sigma _i^z$ to represent the state of a qubit *i*—when $\sigma_{i}^{z}=1, {\hskip2pt}|\sigma_{i}^{z}\rangle$ represents the qubit *i* in state $|1\rangle$ and when $\sigma_{i}^{z}=-1, {\hskip2pt}|\sigma_{i}^{z}\rangle$ represents the qubit *i* in state $|0\rangle$. The total state of *n* qubits is represented by the basis $|x\rangle = \left| {\sigma _1^z\sigma _2^z...\sigma _n^z} \right\rangle$. $\phi (x) = \sqrt {P(x)}$ where *P*(*x*) is the probability for *x* from the distribution determined by the RBM. The probability of a specific set $x = \{ \sigma _1^z,\sigma _2^z...\sigma _n^z\}$ is:

2 $$P(x) = \frac{{\mathop {\sum}\nolimits_{\{ h\} } {e^{\left( {\mathop {\sum}\nolimits_i {a_i\sigma _i^z} + \mathop {\sum}\nolimits_j {b_jh_j} + \mathop {\sum}\nolimits_{i,j} {w_{ij}\sigma _i^zh_j} } \right)}} }}{{\mathop {\sum}\nolimits_{x\prime } {\mathop {\sum}\nolimits_{\{ h\} } {e^{\left( {\mathop {\sum}\nolimits_i {a_i\sigma _i^{z\prime }} + \mathop {\sum}\nolimits_j {b_jh_j} + \mathop {\sum}\nolimits_{i,j} {w_{ij}\sigma _i^{z\prime }h_j} } \right)}} } }}.$$

**Fig. 1:**
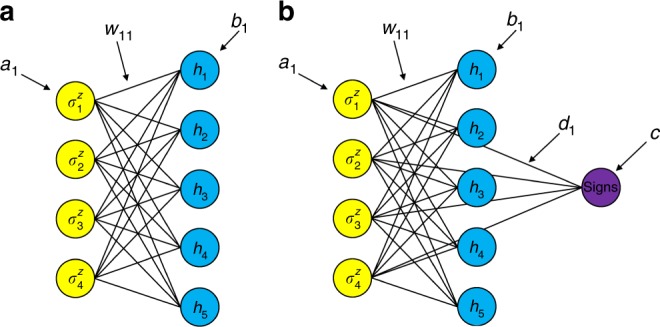
Constructions of restricted Boltzmann machine. **a** The original restricted Boltzmann machine (RBM) structure with visible $\sigma _z^i$ and hidden *h*_*j*_ layers. **b** Improved RBM structure with three layers, visible, hidden and sign. *a*_*i*_, *w*_*ij*_, *b*_*j*_, *d*_*i*_, *c* are trainable weights describing the different connection between layers

Within the above *a*_*i*_ and *b*_*j*_ are trainable weights for units $\sigma _i^z$ and *h*_*j*_. *w*_*ij*_ are trainable weights describing the connections between $\sigma _i^z$ and *h*_*j*_ (see Fig. 1.)

By setting 〈*H*〉 as the objective function of this RBM, we can use the standard gradient decent method to update parameters, effectively minimizing 〈*H*〉 to obtain the ground state energy.

However, previous prescriptions considering the use of RBMs for electronic structure problems have found difficulty as *ϕ*(*x*) can only be non-negative values. We have thus appended an additional layer to the neural network architecture to compensate for the lack of sign features specific to electronic structure problems.

We propose an RBM with three layers. The first layer, *σ*^*z*^, describes the parameters building the wave function. The *h*'s within the second layer are parameters for the coefficients for the wave functions and the third layer *s*, represents the signs associated |*x*〉:

3 $$s(x) = s\left( {\sigma _1^z,\sigma _2^z...\sigma _n^z} \right) = tanh\left( {\mathop {\sum}\limits_i d_i\sigma _i^z + c} \right)$$

The *s* uses a non-linear function *tanh* to classify whether the sign should be positive or negative. Because we have added another function for the coefficients, the distribution is not solely decided by RBM. We also need to add our sign function into the distribution. Within this scheme, *c* is a regulation and *d*_*i*_ are weights for $\sigma _i^z$. (see Fig. 1). Our final objective function, now with $|\phi \rangle = \mathop {\sum}\nolimits_x \phi (x)s(x)|x\rangle$, becomes:

4 $$\langle H\rangle = \frac{{\mathop {\sum}\nolimits_{x,x\prime } {\overline {\phi (x)} \overline {s(x)} \langle x|H|x\prime \rangle \phi (x\prime )s(x\prime )} }}{{\mathop {\sum}\nolimits_x {|\phi (x)s(x)|^2} }}$$

After setting the objective function, the learning procedure is performed by sampling to get the distribution of *ϕ*(*x*) and calculating to get *s*(*x*). We then proceed to calculate the joint distribution determined by *ϕ*(*x*) and *s*(*x*). The gradients are determined by the joint distribution and we use gradient decent method to optimize 〈*H*〉 (see Supplementary Note 1). Calculating the the joint distribution is efficient because *s*(*x*) is only related to *x*.

### Electronic structure Hamiltonian preparation

The electronic structure is represented by *N* single-particle orbitals which can be empty or occupied by a spinless electron:^33^

5 $$\hat H = \mathop {\sum}\limits_{i,j} h_{ij}a_i^\dagger a_j + \frac{1}{2}\mathop {\sum}\limits_{i,j,k,l} h_{ijkl}a_i^\dagger a_j^\dagger a_ka_l.$$

where *h*_*ij*_ and *h*_*ijkl*_ are one and two-electron integrals. In this study we use the minimal basis (STO-3G) to calculate them. $a_j^\dagger$ and *a*_*j*_ are creation and annihilation operators for the orbital *j*.

Equation (5) is then transformed to Pauli matrices representation, which is achieved by the Jordan-Wigner transformation^34^. The final electronic structure Hamiltonian takes the general form with $\sigma _\alpha ^i \in \left\{ {\sigma _x,\sigma _y,\sigma _z,I} \right\}$ where *σ*_*x*_, *σ*_*y*_, *σ*_*z*_ are Pauli matrices and *I* is the identity matrix:^35^

6 $$H = \mathop {\sum}\limits_{i,\alpha } h_\alpha ^i\sigma _\alpha ^i + \mathop {\sum}\limits_{i,j,\alpha ,\beta } h_{\alpha \beta }^{ij}\sigma _\alpha ^i\sigma _\beta ^j + \mathop {\sum}\limits_{i,j,k,\alpha ,\beta ,\gamma } h_{\alpha \beta \gamma }^{ijk}\sigma _\alpha ^i\sigma _\beta ^j\sigma _\gamma ^k + ...$$

### Quantum algorithm to sample Gibbs distribution

We propose a quantum algorithm to sample the distribution determined by RBM. The probability for each combination *y* = {*σ*^*z*^, *h*} can be written as:

7 $$P(y) = \frac{{e^{\mathop {\sum}\nolimits_i {a_i\sigma _i^z + \mathop {\sum}\nolimits_j {b_jh_j} + \mathop {\sum}\nolimits_{i,j} {w_{ij}\sigma _i^zh_j} } }}}{{\mathop {\sum}\nolimits_{y\prime } {e^{\mathop {\sum}\nolimits_i {a_i\sigma _i^{z\prime } + \mathop {\sum}\nolimits_j {b_jh_j^\prime } + \mathop {\sum}\nolimits_{i,j} {w_{ij}\sigma _i^{z\prime }h_j^\prime } } }} }},$$

Instead of *P*(*y*), we try to sample the distribution *Q*(*y*) as:

8 $$Q(y) = \frac{{e^{\frac{1}{k}\left( {\mathop {\sum}\nolimits_i {a_i\sigma _i^z} + \mathop {\sum}\nolimits_j {b_jh_j} + \mathop {\sum}\nolimits_{i,j} {w_{ij}\sigma _i^zh_j} } \right)}}}{{\mathop {\sum}\nolimits_{y\prime } {e^{\frac{1}{k}\left( {\mathop {\sum}\nolimits_i {a_i\sigma _i^{z\prime }} + \mathop {\sum}\nolimits_j {b_jh_j^\prime } + \mathop {\sum}\nolimits_{i,j} {w_{ij}\sigma _i^{z\prime }h_j^\prime } } \right)}} }},$$

where *k* is an adjustable constant with different values for each iteration and is chosen to increase the probability of successful sampling. In our simulation, it is chosen as $O\big( {\mathop {\sum}\nolimits_{i,j} |w_{ij}|} \big)$.

We employed a quantum algorithm to sample the Gibbs distribution from the quantum computer. This algorithm is based on sequential applications of controlled-rotation operations, which tries to calculate a distribution *Q*′(*y*) ≥ *Q*(*y*) with an ancilla qubit showing whether the sampling for *Q*(*y*) is successful^23^.

**Fig. 2:**
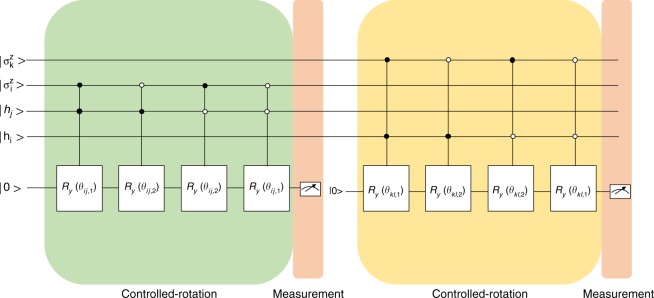
The example circuit for the second step of the controlled-rotation gate approach with measurements

This two-step algorithm uses one system register (with *n* + *m* qubits in use) and one scratchpad register (with one qubit in use) as shown in Fig. 2.

All qubits are initialized as |0〉 at the beginning. The first step is to use *R*_*y*_ gates to get a superposition of all combinations of {*σ*^*z*^, *h*} with $\theta _i = 2arcsin\left( {\sqrt {\frac{{e^{a_i/k}}}{{e^{a_i/k} + e^{ - a_i/k}}}} } \right)$ and $\gamma _j = 2arcsin\left( {\sqrt {\frac{{e^{b_j/k}}}{{e^{b_j/k} + e^{ - b_j/k}}}} } \right)$:

9 $$ \otimes _iR_y(\theta _i)|0_i\rangle \otimes _jR_y(\gamma _j)|0_j\rangle |0\rangle = \sum\nolimits_y\sqrt{O(y)}|y\rangle |0\rangle $$

where $O(y) = \frac{{e^{\mathop {\sum}\nolimits_i a_i\sigma _i^z/k + \mathop {\sum}\nolimits_j b_jh_j/k}}}{{\mathop {\sum}\nolimits_{y\prime } e^{\mathop {\sum}\nolimits_i a_i\sigma _i^{z\prime }/k + \mathop {\sum}\nolimits_j b_jh_j^\prime /k}}}$ and |*y*〉 corresponds to the combination $|y\rangle = |\sigma _1^z...\sigma _n^zh_1...h_m\rangle -$ as before when $h_{j}=1,{\hskip2pt}|\mathrm{h}{{j}}\rangle$ represents the corresponding qubit in state $|1\rangle$ and when $h_{j}=-1,{\hskip2pt}|\mathrm{h}{j}\rangle$ represents the corresponding qubit in state $|0\rangle$.

The second step is to calculate $e^{w_{ij}\sigma _i^zh_j}$. We use controlled-rotation gates to achieve this. The idea of sequential controlled-rotation gates is to check whether the target qubit is in state |0〉 or state |1〉 and then rotate the corresponding angle (Fig. 2). If qubits $\sigma _i^z h_j$ are in |00〉 or |11〉, the ancilla qubit is rotated by *R*_*y*_(*θ*_*ij*,1_) and otherwise by *R*_*y*_(*θ*_*ij*,2_), with $\theta _{ij,1} = 2arcsin\left( {\sqrt {\frac{{e^{w_{ij}/k}}}{{e^{\left| {w_{ij}} \right|/k}}}} } \right)$ and $\theta _{ij,2} = 2arcsin\left( {\sqrt {\frac{{e^{ - w_{ij}/k}}}{{e^{|w_{ij}|/k}}}} } \right)$. Each time after one $e^{w_{ij}\sigma _i^zh_j}$ is calculated, we do a measurement on the ancilla qubit. If it is in |1〉 we continue with a new ancilla qubit initialized in |0〉, otherwise we start over from the beginning (details in Supplementary Note 2).

**Fig. 3:**
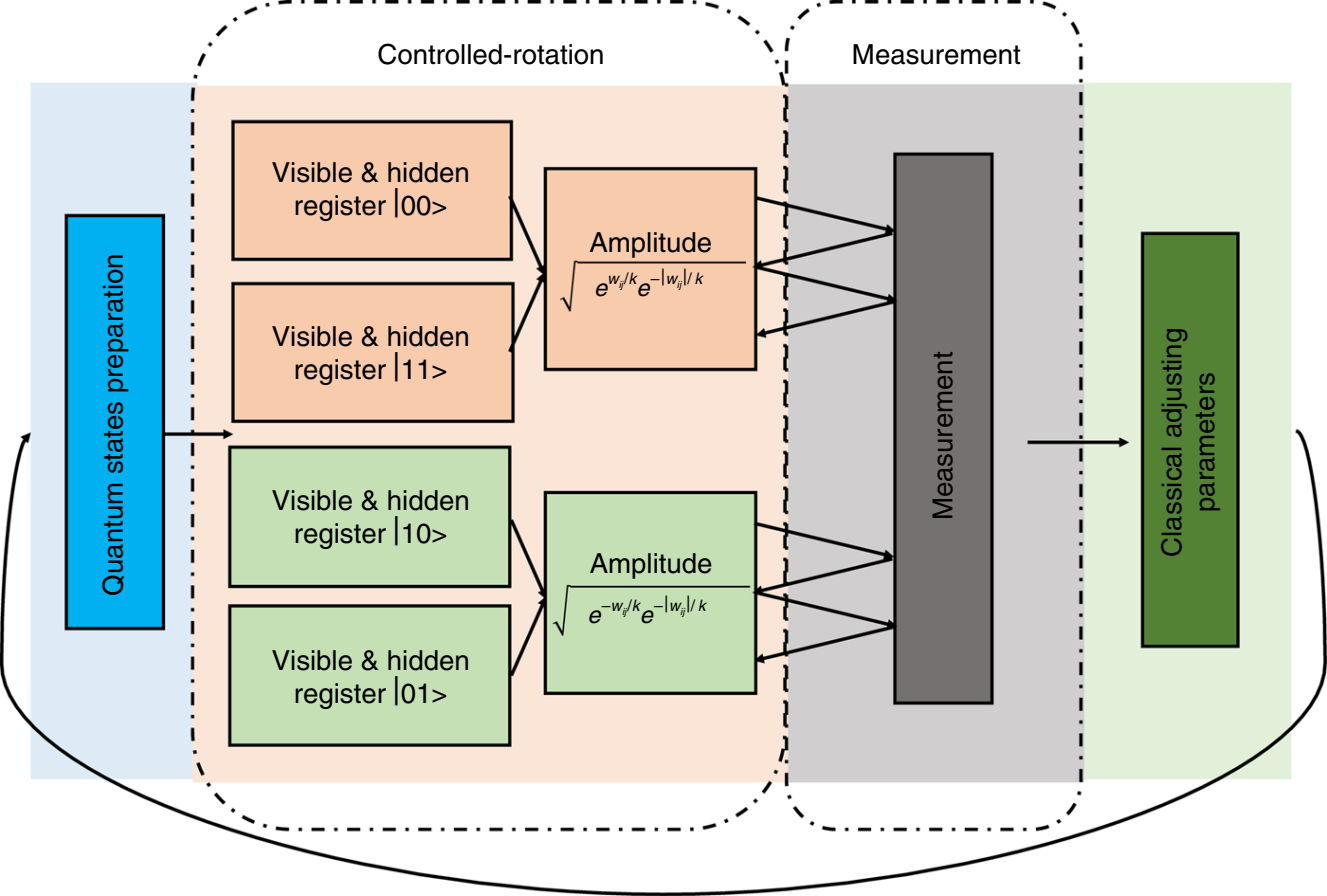
The algorithmic flow chart of the quantum algorithm based on sequential controlled-rotations gates

After we finish all measurements the final states of the first *m* + *n* qubits follow the distribution *Q*(*y*). We just measure the first *n* + *m* qubits of the system register to obtain the probability distribution. After we get the distribution, we calculate all probabilities to the power of *k* and normalize to get the Gibbs distribution (Fig. 3).

The complexity of gates comes to *O*(*mn*) for one sampling and the qubits requirement comes to *O*(*mn*). If considering the reuse of ancilla qubits, the qubits requirements reduce to *O*(*m* + *n*) (see Supplementary Note 4). The probability of one successful sampling has a lower bound $e^{\frac{{ - 1}}{k}\mathop {\sum}\nolimits_{i,j} 2|w_{ij}|}$ and if *k* is set to $O\left( {\mathop {\sum}\nolimits_{i,j} |w_{ij}|} \right)$ it has constant lower bound (see Supplementary Note 3). If *N*_*s*_ is the number of successful sampling to get the distribution, the complexity for one iteration should be *O*(*N*_*s*_*mn*) due to the constant lower bound of successful sampling as well as processing distribution taking *O*(*N*_*s*_). In the meantime, the exact calculation for the distribution has complexity as *O*(2^*m*+*n*^). The only error comes from the error of sampling if not considering noise in the quantum computer.

### Summary of numerical results

**Fig. 4:**
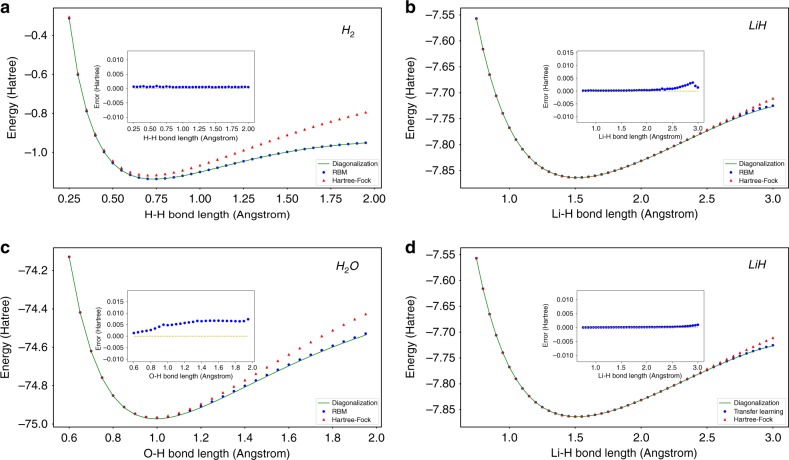
Results of calculating ground state energy of H_2_, LiH and H_2_O. **a**–**c** are the results of H_2_ (*n* = 4, *m* = 8), LiH (*n* = 4, *m* = 8) and H_2_O (*n* = 6, *m* = 6) calculated by our three layer RBM compared with exact diagonalized results of the transformed Hamiltonian. **d** is the result of LiH (*n* = 4, *m* = 8) calculated by the Transfer Learning method. We use STO-3G as basis to compute the molecular integrals for the Hamiltonian. Bond length represents inter-atomic distance for the diatomic molecules and the distance O-H of the optimized equilibrium structure of the water molecule. The data points of RBM are minimum energies of all energies calculated during the whole optimization by sampling

We now present the results derived from our RBM for H_2_, LiH and H_2_O molecules. It can clearly be seen from Fig. 4 that our three layer RBM yields very accurate results comparing to the disorganization of transformed Hamiltonian which is calculated by a finite minimal basis set, STO-3G. Points deviating from the ideal curve are likely due to local minima trapping during the optimization procedure. This can be avoided in the future by implementing optimization methods which include momentum or excitation, increasing the escape probability from any local features of the potential energy surface.

Further discussion about our results should mention instances of transfer learning. Transfer learning is a unique facet of neural network machine learning algorithms describing an instance (engineered or otherwise) where the solution to a problem can inform or assist in the solution to another similar subsequent problem. Given a diatomic Hamiltonian at a specific intermolecular separation, the solution yielding the variational parameters—which are the weighting coefficients of the basis functions—are adequate first approximations to those parameters at a subsequent calculation where the intermolecular separation is a small perturbation to the previous value.

Except for the last point in the Fig. 4d, we use 1/40 of the iterations for the last point in calculations initiated with transferred parameters from previous iterations of each points and still achieve a good result. We also see that the local minimum is avoided if the starting point achieve global minimum.

## Discussion

In conclusion, we present a combined quantum machine learning approach to perform electronic structure calculations. Here, we have a proof of concept and show results for small molecular systems. Screening molecules to accelerate the discovery of new materials for specific application is demanding since the chemical space is very large! For example, it was reported that the total number of possible small organic molecules that populate the ‘chemical space’ exceed 10^60^ ^36,37^. Such an enormous size makes a thorough exploration of chemical space using the traditional electronic structure methods impossible. Moreover, in a recent perspective^38^in Nature Reviews Materials the potential of machine learning algorithms to accelerate the discovery of materials was pointed out. Machine learning algorithms have been used for material screening. For example, out of the GDB-17 data base, consisting of about 166 billion molecular graphs, one can make organic and drug-like molecules with up to 17 atoms and 134 thousand smallest molecules with up to 9 heavy atoms were calculated using hybrid density functional (B3LYP/6-31G(2df,p). Machine learning algorithms trained on these data, were found to predict molecular properties of subsets of these molecules^38–40^.

In the current simulation, H_2_ requires 13 qubits with the number of visible units *n* = 4, the number of hidden units *m* = 8 and additional 1 reusing ancilla qubit. LiH requires 13 qubits with the number of visible units *n* = 4, the number of hidden units *m* = 8 and additional 1 reusing ancilla qubit. H_2_O requires 13 qubits with the number of visible units *n* = 6, the number of hidden units *m* = 6 and additional 1 reusing ancilla qubit. The order of scaling of qubits for the system should be *O*(*m* + *n*) with reusing ancilla qubits. The number of visible units *n* is equal to the number of spin orbitals. The choice of the number of hidden units *m* is normally integer times of *n* which gives us a scaling of *O*(*n*) with reusing ancilla qubits. Thus, the scaling of the qubits increases polynomially with the number of spin orbitals. Also, the complexity of gates *O*(*n*^2^) scales polynomially with the number of spin orbitals while the scaling of classical Machine Learning approaches calculating exact Gibbs distribution is exponential. With the rapid development of larger-scale quantum computers and the possible training of some machine units with the simple dimensional scaling results for electronic structure, quantum machine learning techniques are set to become powerful tools to perform electronic structure calculations and assist in designing new materials for specific applications.

## Methods

### Preparation of the Hamiltonian of H_2_, LiH and H_2_O

We treat H_2_ molecule with 2-electrons in a minimal basis STO-3G and use the Jordan-Wigner transformation^34^. The final Hamiltonian is of 4 qubits. We treat LiH molecule with 4-electrons in a minimal basis STO-3G and use the Jordan-Wigner transformation^34^. We assumed the first two lowest orbitals are occupied by electrons and the the final Hamiltonian is of 4 qubits. We treat H_2_O molecule with 10-electrons in a minimal basis STO-3G, we use Jordan-Wigner transformation^34^. We assume the first four lowest energy orbitals are occupied by electrons and first two highest energy orbitals are not occupied all time. We also use the spin symmetry in^41,42^ to reduce another two qubits. With the reduction of the number of qubits, finally we have 6 qubits Hamiltonian^35,43^. All calculations of integrals in second quantization and transformations of electronic structure are done by OpenFermion^44^ and Psi4^45^.

### Gradient estimation

The two functions *ϕ*(*x*) and *s*(*x*) are both real function. Thus, the gradient for parameter *p*_*k*_ can be estimated as $2\left( {\left\langle {E_{loc}D_{p_k}} \right\rangle - \left\langle {E_{loc}} \right\rangle \left\langle {D_{p_k}} \right\rangle } \right)$ where $E_{loc}(x) = \frac{{\langle x|H|\phi \rangle }}{{\phi (x)s(x)}}$ is so called local energy, $D_{p_k}(x) = \frac{{\partial _{p_k}(\phi (x)s(x))}}{{\phi (x)s(x)}}$. 〈...〉 represents the expectation value of joint distribution determined by *ϕ*(*x*) and *s*(*x*) (details in Supplementary Note 1).

### Implementation details

In our simulation we choose small constant learning rate 0.01 to avoid trapping in local minimum. All parameter are initialized as a random number between (−0.02,0.02). The range of initial random parameter is to avoid gradient vanishing of *tanh*. For each calculation we just need 1 reusing ancilla qubit all the time. Thus, in the simulation, the number of required qubits is *m* + *n* + 1. All calculations do not consider the noise and system error (details in Supplementary Note 5).

## Electronic supplementary material

Supplementary Information

## Data Availability

The data and codes that support the findings of this study are available from the corresponding author upon reasonable request.
