# Supplementary material for: Quantum machine learning for electronic structure calculations
Source: Nat Commun. 2018 Oct 10;9:4195. doi: 10.1038/s41467-018-06598-z (PMC6180079; doi:10.1038/s41467-018-06598-z)
Supplement: Supplementary file 1 — Supplementary Information [file 41467_2018_6598_MOESM1_ESM.pdf]

Quantum Machine Learning for Electronic Structure Calculations Supplementary Information

Rongxin Xia[1] and Sabre Kais *[1,2,3]

[1] *Department of Physics and Astronomy, Purdue University, West Lafayette, IN, 47907 USA*
[2] *Department of Chemistry and Birck Nanotechnology Center, Purdue University, West Lafayette, IN 47907 USA*
[3] *Santa Fe Institute, 1399 Hyde Park Rd, Santa Fe, NM 87501*

*kais@purdue.edu

Supplementary Note 1

Derivation of the gradient

For an electronic structure Hamiltonian prepared by second quantization and Jordan-Wigner transformation[1], H, and a trial wave function, $|\phi\rangle = \sum_x \phi(x)s(x)|x\rangle$, the expectation value can be written as[2]:

$$\langle H \rangle = \frac{\langle \phi | H | \phi \rangle}{\langle \phi | \phi \rangle} = \frac{\sum_{x,x'} \overline{\phi(x)s(x)} \langle x | H | x' \rangle \phi(x') s(x')}{\sum_x |\phi(x)s(x)|^2} \tag{1}$$

x is a combination of $\{\sigma_1^z, \sigma_2^z ... \sigma_n^z\}$ and $|x\rangle = |\sigma_1^z \sigma_2^z ... \sigma_n^z\rangle$.

If we set $\Phi(x) = \phi(x)s(x)$, because $\phi(x)$ and $s(x)$ are all real value functions, then the gradient can be calculated as[2, 3]:

$$\begin{aligned} \partial_{p_k} \langle H \rangle = & \frac{\sum_x (\partial_{p_k} \Phi(x)) \langle x | H | \phi \rangle + \sum_x \langle \phi | H | x \rangle (\partial_{p_k} \Phi(x))}{\sum_x |\Phi(x)|^2} \\ & - \frac{\sum_x \Phi(x) \langle x | H | \phi \rangle}{\sum_x |\Phi(x)|^2} \frac{\sum_x ((\partial_{p_k} \Phi(x)) \Phi(x) + \Phi(x) \partial_{p_k} \Phi(x))}{\sum_x |\Phi(x)|^2} \end{aligned} \tag{2}$$

If we set $E_{loc}(x) = \frac{\langle x|H|\phi\rangle}{\Phi(x)}$ and $D_{p_k}(x) = \frac{\partial_{p_k}\Phi(x)}{\Phi(x)}$, the gradient can be written as[2]:

$$\begin{aligned} \partial_{p_k} \langle H \rangle = & \frac{\sum_x D_{p_k}(x) E_{loc}(x) |\Phi(x)|^2 + \sum_x E_{loc}(x) D_{p_k}(x) |\Phi(x)|^2}{\sum_x |\Phi(x)|^2} \\ & - \frac{\sum_x |\Phi(x)|^2 E_{loc}(x)}{\sum_x |\Phi(x)|^2} \frac{\sum_x (D_{p_k}(x) + D_{p_k}(x)) |\Phi(x)|^2}{\sum_x |\Phi(x)|^2} \\ = & 2 \langle E_{loc} D_{p_k} \rangle - 2 \langle E_{loc} \rangle \langle D_{p_k} \rangle \end{aligned} \tag{3}$$

where $\langle ... \rangle$ represent the expectation value of distribution determined by $\Phi(x)$. $\langle x|H|\phi\rangle = \langle \phi|H|x\rangle$ for that H is a real symmetric matrix due to Jordan-Wigner transformation.

p_k is the parameters a_i, b_j, w_{ij}, d_i, c for k_{th} iterations. Thus we have[2]:

$$\begin{aligned} D_{a_i}(x) &= \frac{1}{2}\sigma_i^z - \frac{1}{2}\langle \sigma_i^z \rangle_{RBM}, \\ D_{b_j}(x) &= \frac{1}{2}tanh(\theta_j) - \frac{1}{2}\langle h_j \rangle_{RBM}, \\ D_{w_{ij}}(x) &= \frac{1}{2}tanh(\theta_j)\sigma_i^z - \frac{1}{2}\langle \sigma_i^z h_j \rangle_{RBM}, \\ D_c(x) &= 1/s(x) - s(x), \\ D_{d_i}(x) &= \sigma_i^z(1/s(x) - s(x)), \end{aligned} \tag{4}$$

where $\theta_j = \sum_i w_{ij}\sigma_z^i + b_j$. $\langle ... \rangle_{RBM}$ represents the distribution determined solely by RBM. We do not need to calculate the second term of D_{a_i}, D_{b_i} and $D_{w_{ij}}$ for that they will be cancelled when calculating the gradient $\partial_{p_k}\langle H \rangle$. We use the gradient decent method to optimize our RBM, yielding the global minimum corresponding to the ground energy.

$$p_{k+1} = p_k - \alpha_k \partial_{p_k} \langle H \rangle \tag{5}$$

Where α_k is the learning rate for k_{th} iteration, controlling the convergence rate. We can continue iterating until we reach the maximum number of iterations. The gradient is estimated by the distribution calculated by sampling.

Supplementary Note 2

Sequential applications of controlled-rotation algorithm

The probability for each combination $y = \{\sigma^z,\ h\}$ can be written as:

$$P(y) = \frac{e^{\sum_i a_i\sigma_i^z + \sum_j b_j h_j + \sum_{i,j} w_{ij}\sigma_i^z h_j}}{\sum_{y'} e^{\sum_i a_i\sigma_i^{z\prime} + \sum_j b_j h_j' + \sum_{i,j} w_{ij}\sigma_i^{z\prime} h_j'}} \tag{6}$$

However, we do not directly calculate the $P(y)$ but we do some modification on $P(y)$ to increase the successful probability of our algorithm. We calculate $Q(y) = \frac{e^{\sum_i a_i\sigma_i^z/k + \sum_j b_j h_j/k + \sum_{i,j} w_{ij}\sigma_i^z h_j/k}}{\sum_{y'} e^{\sum_i a_i\sigma_i^{z\prime}/k + \sum_j b_j h_j'/k + \sum_{i,j} w_{ij}\sigma_i^{z\prime} h_j'/k}}$ where k is a large number to increase the successful probability of our measurements.

First we use R_y gate to achieve a superposition of all possible σ_z and h. The system qubits and the ancilla qubit are initialized at state $|0\rangle$.

$$\otimes_i R_y(2arcsin(\sqrt{\frac{e^{a_i/k}}{e^{a_i/k} + e^{-a_i/k}}}))|0_i\rangle \otimes_j R_y(2arcsin(\sqrt{\frac{e^{b_j/k}}{e^{b_j/k} + e^{-b_j/k}}}))|0_j\rangle|0\rangle = \sum_y O(y)|y\rangle|0\rangle \tag{7}$$

where $O(y) = \frac{e^{\sum_i a_i\sigma_i^z/k + \sum_j b_j h_j/k}}{\sum_{y'} e^{\sum_i a_i\sigma_i^{z\prime}/k + \sum_j b_j h_j'/k}}$ and $|y\rangle = |\sigma_1^z..\sigma_n^z h_1...h_m\rangle$.

The next step is to calculate each term of $e^{\sum_{i,j} w_{ij}\sigma_i^z h_j}$, which is achieved by controlled rotations gates. The idea is, for each time controlled rotation, we calculate two angles $\theta_{ij,1} = 2arcsin(\sqrt{e^{w_{ij}/k}e^{-|w_{ij}|/k}})$ and $\theta_{ij,2} = 2arcsin(\sqrt{e^{-w_{ij}/k}e^{-|w_{ij}|/k}})$. We use controlled-rotation $CR_y(\theta_{ij,1})$ and $CR_y(\theta_{ij,2})$ which are controlled by combination of σ_i^z, h_j as working qubits to do rotation on the ancilla qubit. The controlled rotation is to check the working qubits and then do the corresponding rotation $\theta_{ij,1}$ or $\theta_{ij,2}$.

All controlled rotation gates can be expressed as below:

$$\begin{aligned}
CR_{w_{ij},1} &= C_{\sigma_i^z,h_j} \otimes R_y(2arcsin(\sqrt{e^{w_{ij}/k}e^{-|w_{ij}|/k}})) + (D_{\sigma_i^z,h_j} + E_{\sigma_i^z,h_j} + F_{\sigma_i^z,h_j}) \otimes I \\
CR_{w_{ij},2} &= D_{\sigma_i^z,h_j} \otimes R_y(2arcsin(\sqrt{e^{-w_{ij}/k}e^{-|w_{ij}|/k}})) + (C_{\sigma_i^z,h_j} + E_{\sigma_i^z,h_j} + F_{\sigma_i^z,h_j}) \otimes I \\
CR_{w_{ij},3} &= E_{\sigma_i^z,h_j} \otimes R_y(2arcsin(\sqrt{e^{-w_{ij}/k}e^{-|w_{ij}|/k}})) + (C_{\sigma_i^z,h_j} + D_{\sigma_i^z,h_j} + F_{\sigma_i^z,h_j}) \otimes I \\
CR_{w_{ij},4} &= F_{\sigma_i^z,h_j} \otimes R_y(2arcsin(\sqrt{e^{w_{ij}/k}e^{-|w_{ij}|/k}})) + (C_{\sigma_i^z,h_j} + D_{\sigma_i^z,h_j} + E_{\sigma_i^z,h_j}) \otimes I
\end{aligned} \tag{8}$$

where $C_{\sigma_i^z,h_j} = B_{\sigma_i^z} \otimes B_{h_j}$, $D_{\sigma_i^z,h_j} = A_{\sigma_i^z} \otimes B_{h_j}$, $E_{\sigma_i^z,h_j} = B_{\sigma_i^z} \otimes A_{h_j}$, $F_{\sigma_i^z,h_j} = A_{\sigma_i^z} \otimes A_{h_j}$ and

$$A = \begin{bmatrix} 1 & 0 \\ 0 & 0 \end{bmatrix} B = \begin{bmatrix} 0 & 0 \\ 0 & 1 \end{bmatrix}$$

Between the calculation of two w_{ij}, we need to do a measurement on the ancilla qubit to make sure the state of system qubits collapse to the wanted state. Measuring ancilla qubit in $|1\rangle$ means the state of system qubits collapse to the wanted state as we initialize the ancilla qubit in $|0\rangle$.

We then do the measurement, if and only if the ancilla qubit is in $|1\rangle$ we continue with a new ancilla qubit initialized in $|0\rangle$, otherwise we start from beginning. The probability of success is very large since we choose k as a large number.

After we finish all measurements, the distribution should be $Q(y)$. We just measure the first $n+m$ qubits of the system register to obtain the probability distribution. After we get the distribution, we calculate all probabilities to the power of k and normalize to get the Gibbs distribution.

Supplementary Note 3

Lower bound of successful sampling

The successful probability P can be written as:

$$P = \frac{\sum_{\sigma_z,h} e^{\frac{1}{k}(\sum_i a_i\sigma_i^z + \sum_j b_j h_j + \sum_{i,j} w_{ij}\sigma_i^z h_j)}}{\sum_{\sigma_z,h} e^{\frac{1}{k}(\sum_i a_i\sigma_i^z + \sum_j b_j h_j)} e^{\frac{1}{k}(\sum_{i,j}|w_{ij}|)}} \geq \frac{e^{\frac{-1}{k}(\sum_{i,j}|w_{ij}|)}}{e^{\frac{1}{k}(\sum_{i,j}|w_{ij}|)}} = \frac{1}{e^{\frac{1}{k}(\sum_{i,j}2|w_{ij}|)}} \tag{9}$$

If we choose $k = O(\sum_{i,j}|w_{ij}|)$, we have $P \geq \frac{1}{e^{O(1)}}$ which means the lower bound of probability of successful sampling is a constant. In the simulation, we choose $k = max(\frac{1}{2}\sum_{i,j}|w_{ij}|, 1)$ because larger k introduces larger sampling errors. This particular choice of k gives us lower bound of success as e^{-4}. But in numerical simulation, the probability is much larger than e^{-4}, see **Supplementary Note 5**.

Supplementary Note 4

Complexity and error of the algorithm

For a $C^2(U)$ conditioned by $|11\rangle$, it can be decomposed as the below[4]:

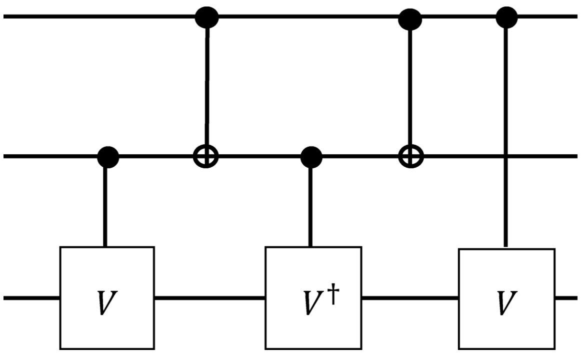

Supplementary Figure 1: The decomposition of the $C^2(U)$ gate.

where $V^2 = U$. In our algorithm, $U = R_y(\theta)$, thus we can choose $V = R_y(\theta/2)$ to achieve the decomposition. $C^2(U)$ conditioned by $|00\rangle$, $|10\rangle$ or $|01\rangle$ can be achieved by adding X gates on controlling qubits. For each w_{ij}, we have 4 $C^2(U)$ which means the gates complexity scales to $O(mn)$ and the number of qubits for our algorithms scales to $O(mn)$, which can be reduced to $O(m+n)$ if considering qubit reuse. Because the lower bound of probability of successful sampling is constant, if the number of successful sampling is N_s, the complexity for each iteration is $O(N_s mn)$. The only error comes from the error of sampling if not considering noise in the quantum computer.

Supplementary Note 5

Implementation details for H_2, LiH and H_2O

Here we present the probabilities of successful sampling when calculating H_2, LiH and H_2O for bond length equals to 1.75 Angstrom.

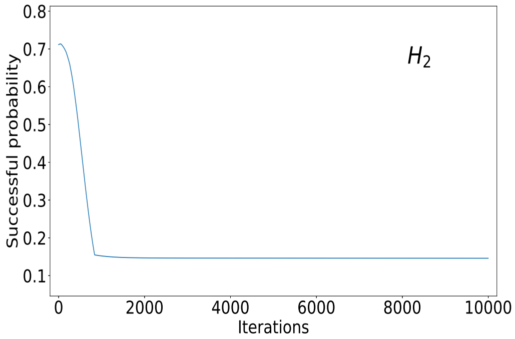

a

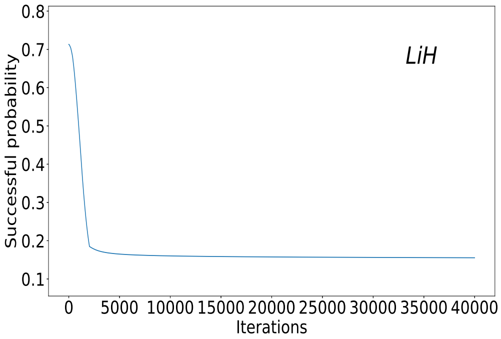

b

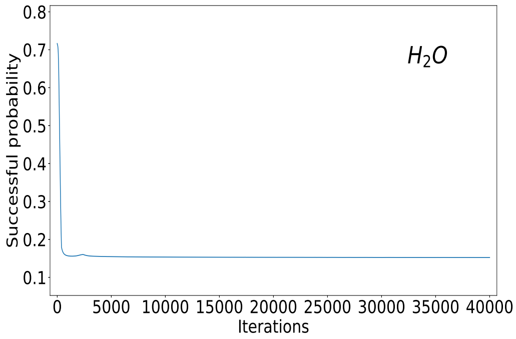

c

Supplementary Figure 2: The probability of successful sampling during the optimization. **a** Optimization procedure for H_2. **b** Optimization procedure for LiH. **c** Optimization procedure for H_2O.

Here we present the the changes of energy during optimization when calculating H_2, LiH and H_2O for bond length equals to 1.75 Angstrom..

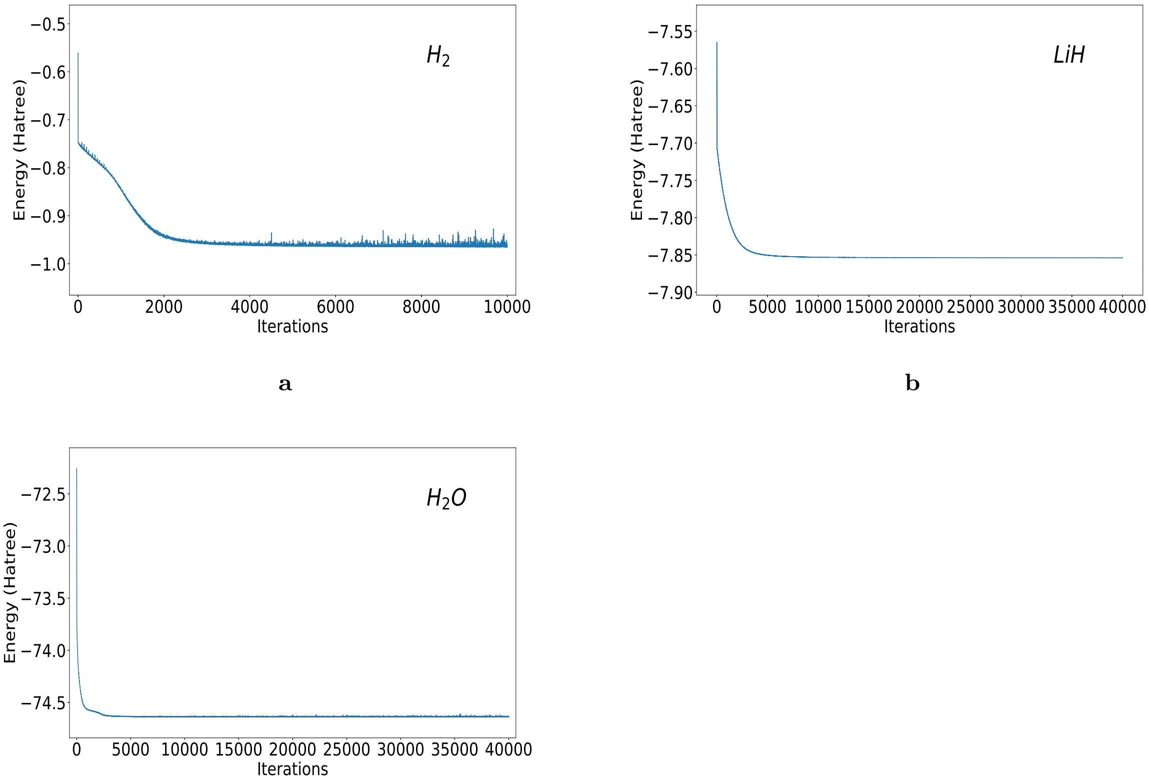

a **b** **c**

Supplementary Figure 3: The energies calculated by RBM during the optimization. **a** Optimization procedure for H_2. **b** Optimization procedure for LiH. **c** Optimization procedure for H_2O.

The distribution we want to sampling for the quantum algorithm is:

$$Q(y) = \frac{e^{\sum_i a_i \sigma_i^z/k + \sum_j b_j h_j/k + \sum_{i,j} w_{ij}\sigma_i^z h_j/k}}{\sum_{y'} e^{\sum_i a_i \sigma_i^{z\prime}/k + \sum_j b_j h_j'/k + \sum_{i,j} w_{ij}\sigma_i^{z\prime} h_j'/k}} \tag{10}$$

In our controlled-rotation algorithm, we use a k as regulation to increase the probability of success as the proof in the **Supplementary Note 3**, the lower bound of probability of success would become :

$$\frac{1}{e^{\frac{1}{k}(\sum_{i,j} 2|w_{ij}|)}} \tag{11}$$

Thus, if no regulation ($k = 1$), the probability of success would become $\frac{1}{e^{\sum_{i,j} 2|w_{ij}|}}$ which means we need exponential number of measurements to get enough successful sampling, making no speedup in quantum algorithm.

If we add a regulation of k, in simulation we use $k = \frac{1}{2}\sum_{i,j}|w_{ij}|$, the probability becomes $\frac{1}{e^4}$ which needs constant number of measurements to get enough successful sampling.

After we get the distribution, we need to calculate all distribution to the power of k and normalize to get the wanted distribution. k is a large number, which is around 5 at final for H_2, LiH and H_2O in our simulation. To decrease the errors in calculating power of k, we have to increase the number of sampling for

our quantum algorithm when k is large, which requires large number of sampling and may not be efficient. In the **Supplementary Figure 3**, we can see that at the final procedure of optimization, the fluctuation is very large due to large k, which can be decreased by increasing the number of sampling. Because we investigated small molecule system H_2, LiH and H_2O, k is not very large and the quantum algorithm is efficient. For large k, our quantum algorithm may require large sampling.

References

[1] Fradkin, E. Jordan-wigner transformation for quantum-spin systems in two dimensions and fractional statistics. *Physical review letters* **63**, 322 (1989).

[2] Carleo, G. *et al.* Neural-network quantum states. *Bulletin of the American Physical Society* (2018).

[3] Carleo, G. & Troyer, M. Solving the quantum many-body problem with artificial neural networks. *Science* **355**, 602–606 (2017).

[4] Nielsen, M. A. & Chuang, I. L. *Quantum computation and quantum information* (Cambridge University Press, Cambridge, 2000).
